# Presence and diversity of Chlamydiae bacteria in *Spinturnix myoti*, an ectoparasite of bats

**DOI:** 10.1051/parasite/2020052

**Published:** 2020-11-02

**Authors:** Kevin Thiévent, Tamara Szentiványi, Sébastien Aeby, Olivier Glaizot, Philippe Christe, Gilbert Greub

**Affiliations:** 1 Center for Research on Intracellular Bacteria (CRIB), Institute of Microbiology, University Hospital Center and University of Lausanne 1011 Lausanne Switzerland; 2 Museum of Zoology 1005 Lausanne Switzerland; 3 Department of Ecology and Evolution, University of Lausanne 1015 Lausanne Switzerland

**Keywords:** Chlamydiae, Mites, Bats, Vector, Epidemiology, Phylogenetic analysis

## Abstract

*Chlamydia* spp. and *Chlamydia*-like organisms are able to infect vertebrates such as mammals, reptiles and birds, but also arthropods and protozoans. Since they have been detected in bats and bat feces, we expected Chlamydiae bacteria to also be present in the mite *Spinturnix myoti*, an ectoparasite of mouse-eared bats (*Myotis* spp.). The prevalence of Chlamydiales in 88 *S. myoti* was 57.95% and significantly depended on bat host species. In addition, the prevalence was significantly different between bat species living in sympatry or in allopatry. While there was uninterpretable sequencing for 16 samples, eight showed best BLAST hit identities lower than 92.5% and thus corresponded to new family-level lineages according to the established taxonomy cut-off. The four remaining sequences exhibited best BLAST hit identities ranging from 94.2 to 97.4% and were taxonomically assigned to three different family-level lineages, with two of them belonging to the Parachlamydiaceae, one to the Simkaniaceae, and one to the Chlamydiaceae. These results highlighted for the first time the presence of *Chlamydia*-like organisms and the possible zoonotic origin of *Chlamydia* sp. in *S. myoti* ectoparasites of bats, and therefore suggest that these ectoparasites may play a role in maintaining and/or transmitting members of the Chlamydiae phylum within *Myotis* spp. bat populations. Our results further highlight that the wide diversity of bacteria belonging to the Chlamydiae phylum is largely underestimated.

## Abstract (fr)

Les *Chlamydia* spp. et les organismes apparentés aux *Chlamydia* sont capables d’infecter des vertébrés tels que les mammifères, les reptiles et les oiseaux mais aussi des arthropodes et des protozoaires. Puisqu’elles ont été détectées dans des chauves-souris et des excréments de chauves-souris, nous nous attendions à ce que les bactéries du phylum Chlamydiae soient également présentes dans des *Spinturnix myoti*, des acariens ectoparasites de chauves-souris du groupe des murins (*Myotis* spp.). La prévalence des Chlamydiales dans 88 *S. myoti* était de 57,95 % et dépendait de manière significative des espèces hôtes de chauves-souris. De plus, la prévalence était significativement différente entre les chauves-souris vivant en sympatrie ou en allopatrie. Alors qu’il y avait un séquençage ininterprétable pour 16 échantillons, huit présentaient des résultats d’analyse de type de type BLAST avec une similarité inférieure à 92.5% et à 92,5 % et correspondaient donc à de nouvelles familles selon les seuils utilisés en taxonomie par les chlamydiologistes. Les quatre séquences restantes présentaient des résultats BLAST allant de 94,2 à 97,4 % et ont été taxonomiquement attribuées à trois familles ; deux d’entre elles appartenant aux Parachlamydiaceae, une aux Simkaniaceae et enfin une aux Chlamydiaceae. Ces résultats ont mis en évidence pour la première fois la présence d’organismes de type *Chlamydia* mais aussi d’organisme pouvant amener à des zoonoses tel que *Chlamydia* sp. chez *Spinturnix* myoti, un ectoparasite de chauves-souris. Ces résultats suggèrent donc que ces ectoparasites pourraient jouer un rôle dans le maintien et/ou la transmission des membres de l’embranchement des Chlamydiae au sein des populations de chauves-souris du genre *Myotis*. Nos résultats soulignent en outre que la grande diversité des bactéries appartenant à l’embranchement des Chlamydiae est largement sous-estimée.

## Introduction

Chlamydiae are obligate intracellular bacteria known to cause medically and economically important infectious diseases. There are nine recognized families of Chlamydiae within the Chlamydiales order: Parachlamydiaceae, Criblamydiaceae, Rhabdochlamydiaceae, Waddliaceae, Simkaniaceae, Piscichlamydiaceae, Clavichlamydiaceae, Parilichlamydiaceae and Chlamydiaceae [16, 21, 57]. Chlamydiaceae includes two well-known human pathogens, *Chlamydia trachomatis* and *C. pneumoniae*, but also includes other animal pathogens responsible for zoonotic infections such as *C. psittaci* and *C. abortus* [5, 30], the causative agents of psittacosis and sheep abortion, respectively. *Chlamydia trachomatis* is responsible for sexually transmitted infections worldwide but also causes trachoma, a blinding disease in developing countries [2, 35, 54], while *Chlamydia pneumoniae* is an agent responsible for respiratory infections [37]. While members of the Chlamydiaceae family are known to be human and/or animal pathogens and are thus highly studied, the role of bacteria belonging to the eight other family-level lineages (also called *Chlamydia*-like organisms (CLOs)) requires further investigations. *Waddlia chondrophila*, a member of the Waddliaceae family, has recently been associated with miscarriage and tubal infertility [3, 4, 60], while *Simkania negevensis*, *Rhabdochlamydia* spp. and *Parachlamydia* spp. have been associated with respiratory infections [15, 24, 27, 36, 39].

Chlamydiae have been found in a wide variety of environmental samples such as water and soil, but also in different organisms such as mammals, reptiles and birds, as well as in arthropods and protozoans [30, 40, 43, 55]. Recently, CLOs and other members of the Chlamydiaceae family have been discovered in bats. Two fruit bat species were found to harbor bacteria from the Waddliaceae family, with *Waddlia malaysiensis* found in the urine of *Eonycteris spelaea* (Chiroptera: Pteropodidae) in Peninsular Malaysia [13] and *Waddlia cocoyoc* found in skin biopsies of *Artibeus intermedius* (Chiroptera: Phyllostomidae) in Mexico [41]. A recent study found that more than 50% of the feces samples of the bat *Myotis daubentonii* (Chiroptera: Vespertilionidae) carried members of the Chlamydiae phylum in Finland, with most of the positive samples belonging to two main families, the Rhabdochlamydiaceae and the Chlamydiaceae [29]. These studies suggested that bats may act as reservoirs for *Chlamydia* and CLOs, in addition to other pathogens such as *Bartonella* [59], Ebola or SARS [10, 26]. Indeed, colonial habits of bats, in particular during the reproductive season when females aggregate in huge numbers to form nursery colonies, make them particularly susceptible to pathogens and parasites [11]. Although bats seem to be colonized by various members of the Chlamydiae phylum, whether these strict intracellular bacteria are transmitted between bats is yet unknown. However, ectoparasites of bats may be suitable vector candidates for Chlamydiae bacteria.

*Spinturnix* mites are obligate hematophagous ectoparasites of bats and are found on the membranous regions of their host’s body, mainly the wing membranes [47]. *Spinturnix* mites are not able to survive away from their host more than a few hours [23] and are viviparous, a life history strategy that has been proposed to facilitate vertical pathogen transmission [11]. Thus, their ecology and feeding biology makes them good candidates to transmit and maintain Chlamydiae within and between bat populations.

The aim of this study was to evaluate the distribution and diversity of Chlamydiae in *Spinturnix* mites and to reveal their reservoir or vectorial potential for Chlamydiae in bats. To do so, we first screened *Spinturnix myoti*, a species that is specialized on mouse-eared bats (*Myotis* spp.), for the presence of Chlamydiae. We then compared the Chlamydiae prevalence between three different *Myotis* bat species on which mites were collected, and between the six different countries where they were collected. Finally, some of the 16S rRNA genes of positive results were sequenced and submitted to a BLAST analysis.

## Methods

### *Spinturnix* sampling and DNA purification

Bats were captured and *Spinturnix* ectoparasites were collected in 1998, 2005 and 2006 before the Nagoya convention (2010), and thus no specific authorizations were needed. Ectoparasites were placed in 98% ethanol and kept at −20 °C until further processing. Collection sites were located in North Africa, such as Tunisia and Morocco, as well as in Europe, including France, Italy, Spain and Switzerland. *Spinturnix* mites were identified using different morphological keys [18, 19, 49]. *Spinturnix myoti* specimens were retrieved from the lesser and the greater mouse-eared bats, respectively *M. blythii* (Italy (Piemonte), Switzerland (Valais)) and *M. myotis* (Italy (Piemonte), Spain (Andalousia, La Fajara), Switzerland (Jura, Valais, Vaud)) and from the Maghreb mouse-eared bat *Myotis punicus* (France (Corsica), Italy (Sardinia), Morocco, Tunisia). *Myotis myotis* and *M. blythii* live in sympatry throughout Europe and frequently form mixed species colonies (here, Piemonte and Valais). These two species have never been found in sympatry with *M. punicus*. DNA extraction from each sample was performed using a standard proteinase K-phenol chloroform method [48].

### Pan-Chlamydiales real-time PCR and sequencing

A real-time quantitative PCR specific to Chlamydiales performed with a StepOne Plus real-time PCR system (Applied Biosystems, Zug, Switzerland) was used to detect Chlamydiales DNA [37]. Specific forward primer panChl16F2, 5′–CCGCCAACACTGGGACT–3′ (the underlined nucleotides represent locked nucleic acids) and reverse primer panChl16R2, 5′–GGAGTTAGCCGGTGCTTCTTTAC–3′, amplified a DNA fragment of about 200 bp (size is species-dependent) belonging to the Chlamydiales 16S ribosomal RNA-encoding gene, and these fragments were detected using a pan-Chlamydiales specific probe, 5′–FAM [6-carboxfluorescein]-CTACGGGAGGCTGCAGTCGAGAATC-BHQ1 [black hole quencher 1]–3′. Amplifications were performed in a final volume of 20 μL with iTaq Universal Probes Supermix with ROX (Bio-Rad, Reinach, Switzerland), 0.1 μM of each primer and of the probe, and with 5 μL of sample DNA. DNA was amplified after initial denaturation and activation of 3 min at 95 °C for 40 cycles with denaturation, annealing and extension occurring at 95 °C, 67 °C and 72 °C, respectively and each step lasting 15 s. Standard curves were built using a serial dilution of positive control plasmids (ten-fold diluted from 10^5^ to 5 copies). Each *Spinturnix* DNA sample was tested in duplicate in 96-well plates, along with standard dilutions in duplicate, two negative controls and two extraction controls. Only samples with a threshold cycle value (*C*_*t*_) smaller than 35 were sent to Microsynth (Balgach, Switzerland) for Sanger sequencing, since a *C*_*t*_ of 35 is the observed threshold for amplicon sequencing we documented in our laboratory. Although they were not sent for sequence analysis, the samples with *C*_*t*_ higher than 35 were also considered positive since the PCR used in this study was highly specific [37].

### Phylogeny

The partial 16S rRNA regions sequenced here in addition to referenced sequences of the 16S rRNA genes of different Chlamydiales and an outgroup taxa (*Opitutus terrae*, a member of the order Verrucomicrobiales*,* which has previously been used as an outgroup of all Chlamydiales [50]) were aligned using the MUSCLE plug-in [20] with Geneious software [34]. Using this alignment and the MrBayes plug-in [32], we built a Bayesian posterior-probability consensus tree with a total chain length of 1,100,000 and a burn-in length of 11,000, as previously described [29].

### GenBank accession numbers

Assigned accession number for the partial 16S rRNA sequences amplified from *S. myoti* deposited in GenBank are MT844007–MT844018.

### Statistical analysis

Using a general linear model (GLM) with a binomial distribution, prevalence of Chlamydiae within *Spinturnix* was first compared between *S. myoti* males and females. In this analysis, the proportion of positive samples was set as the response variable, *S. myoti* sex was set as fixed factors and the model was weighted by the total number of samples per group (*N*).

Using another GLM with a binomial distribution, prevalence of Chlamydiae within *Spinturnix* was then compared between the bat species on which they were collected. In this analysis, the proportion of positive samples was set as the response variable, the host species were set as fixed factors and the model was weighted by the total number of samples per group (*N*).

As bats were not always found in sympatry, we compared in a second step the Chlamydiae prevalence between the collection sites (i.e. the country where bats were collected) within each bat species independently. To do this, we ran for each bat species a GLM with a binomial distribution and with the proportion of positive *Spinturnix* samples set as the response variable, and the country as a fixed factor.

Third, using a GLM with a binomial distribution, we compared the prevalence between *Spinturnix* mites collected on *M. myotis* and *M. blythii* when these later were found in sympatry (in Piemonte (Italy) and Valais (Switzerland)). The host bat species and the collection site were set as a fixed factor with the proportion of positive results set as response variable.

Finally, in order to get a better idea of the role of *Spinturnix* mites in the transmission of Chlamydiae between bats, we tested whether the fact of living or not in sympatry affected the Chlamydiae prevalence in the *Spinturnix* mites. To do so, we selected the data from the *Spinturnix* collected on *M. myotis* since this was the only bat species found to live both alone or in sympatry. Prevalences of *Spinturnix* between *M. myotis* living in sympatry or not were compared using a GLM with a binomial distribution.

All analyses and graphs of prevalence were performed with R software, version 3.5.2 [44] and its interface Rstudio, version 1.1.463 [46]. Significance of the effects were evaluated using the ANOVA function of the R package “car” [22].

## Results

### Chlamydiae prevalence in *Spinturnix* mites

**Figure 1:**
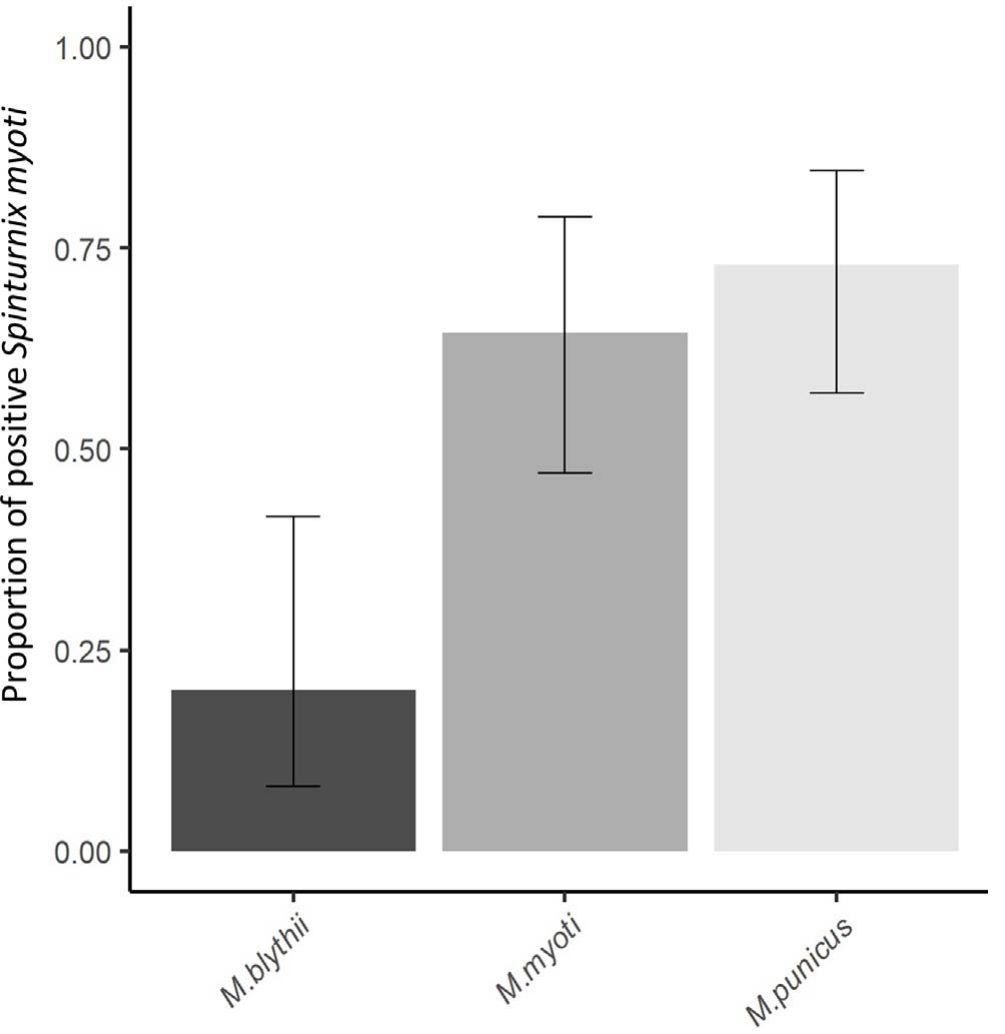
Prevalence of *Chlamydiae* in *Spinturnix myoti* as a function of their host species. Bars represent the 95% confidence intervals.

Of the 88 *Spinturnix myoti*, 57.95% were positive for the presence of Chlamydiae. *Spinturnix myoti* males and females were equally infected by Chlamydiae with 53.33% positive females and 57.97% positive males (*χ*^2^ = 0.002, *df* = 1, *p* = 0.97, Table 1). The Chlamydiae prevalence in *S. myoti* significantly varied between the host bat species with 20% (95% confidence intervals (CI); 8.1–41.6%) *Myotis blythii*, 64.5% (95% CI; 46.9–78.9%) *M. myotis*, and 73% (95% CI; 57–84.6%) *M. punicus* being positive for Chlamydiae (*χ*^2^ = 16.2, *df* = 2, *p* < 0.001) (Fig. 1).

**Table 1:** Summary of Chlamydiae prevalence results as a function of sex and life stage of S. myoti and of bat host species and collection sites.

	Number of tested *S. myoti*	Number of infected *S. myoti*	Chlamydiae prevalence (%)
Sex and life stage of *S. myoti*			
Male	69	40	57.9
Adult	55	38	69.1
Deutonymph	9	2	22.2
Stage undetermined	5	0	0
Female	15	8	53.3
Adult	10	6	60
Stage undetermined	5	2	40
Sex undetermined	4	3	75
Adult	2	2	100
Deutonymph	2	1	50
Bat species and collection sites			
*Myotis punicus*	37	27	72.9
Corsica	12	10	83.3
Sardinia	15	12	80
Morocco	7	3	42.9
Tunisia	3	2	66.7
*Myotis myotis*	31	20	64.5
Spain	10	10	100
Italy	5	2	40
Switzerland	16	8	50
*Myotis blythii*	20	4	20.0
Italy	7	2	28.6
Switzerland	13	2	15.3
Total	88	51	57.95

The results of Chlamydiae prevalence are summarized in Table 1. The Chlamydiae prevalence in *S. myoti* found on *M. punicus* did not vary significantly between the different countries where the mites were collected (*χ*^2^ = 3.98, *df* = 3, *p* = 0.26). Similar results were found for the *S. myoti* collected on *M. blythii*, with no difference in prevalence between mites collected in Switzerland and in Italy (*χ*^2^ = 0.48, *df* = 1, *p* = 0.49). In contrast, the Chlamydiae prevalence in *S. myoti* collected on *M. myotis* significantly varied between the different collection sites (*χ*^2^ = 11.41, *df* = 2, *p* = 0.03), with a prevalence ranging from 40% (Italy) to 100% (Spain).

The Chlamydiae prevalence in *S. myoti* mites was not significantly different between the host bat *M. myotis* and *M. blythii* when these latter were living in sympatry (*χ*^2^ = 0.21, *df* = 1, *p* = 0.64). In fact, 30% (95% CI; 10.8–60.3%) of the *S. myoti* collected on *M. myotis* harbored Chlamydiae, while the prevalence was 20% (95% CI; 8.1–41.6%) for the mites collected on *M. blythii*. In addition, there was no effect of the collection site (*χ*^2^ = 0.95, *df* = 1, *p* = 0.33), nor from the interaction between the host species and the collection sites (*χ*^2^ = 0.01, *df* = 1, *p* = 0.92).

Finally, Chlamydiae prevalence was significantly different between *S. myoti* mites collected on *M. myotis* bats living in sympatry and mites collected on *M. myotis* living in allopatry (*χ*^2^ = 7.66, *df* = 1, *p* = 0.006). In fact, while 80.95% (95% CI; 59.9–92.3%) of the *S. myoti* harbored Chlamydiae when *M. myotis* were found alone, only 30% harbored Chlamydiae when *M. myoti* were living in sympatry with *M. blythii*.

### BLAST and phylogenetic analysis

Of the 51 positive samples, 28 showed *C*_*t*_ values lower than 35 and were thus sequenced. Of these, 16 gave uninterpretable sequences (due to low DNA content or to mixed sequences), and 8 showed best BLAST hit identities smaller than 92.5% and thus correspond to new family-level lineages according to the established taxonomy cut-off [42]. Following this taxonomy cut-off, the 4 remaining samples with best BLAST hits ranging from 94.2% to 97.4% were taxonomically assigned at the family-level lineages with two sequences belonging to the Parachlamydiaceae (GenBank accession numbers MT844011 and MT844016) one to the Simkaniaceae (GenBank accession number MT844014) and one to the Chlamydiaceae (GenBank accession number MT844008).

**Figure 2:**
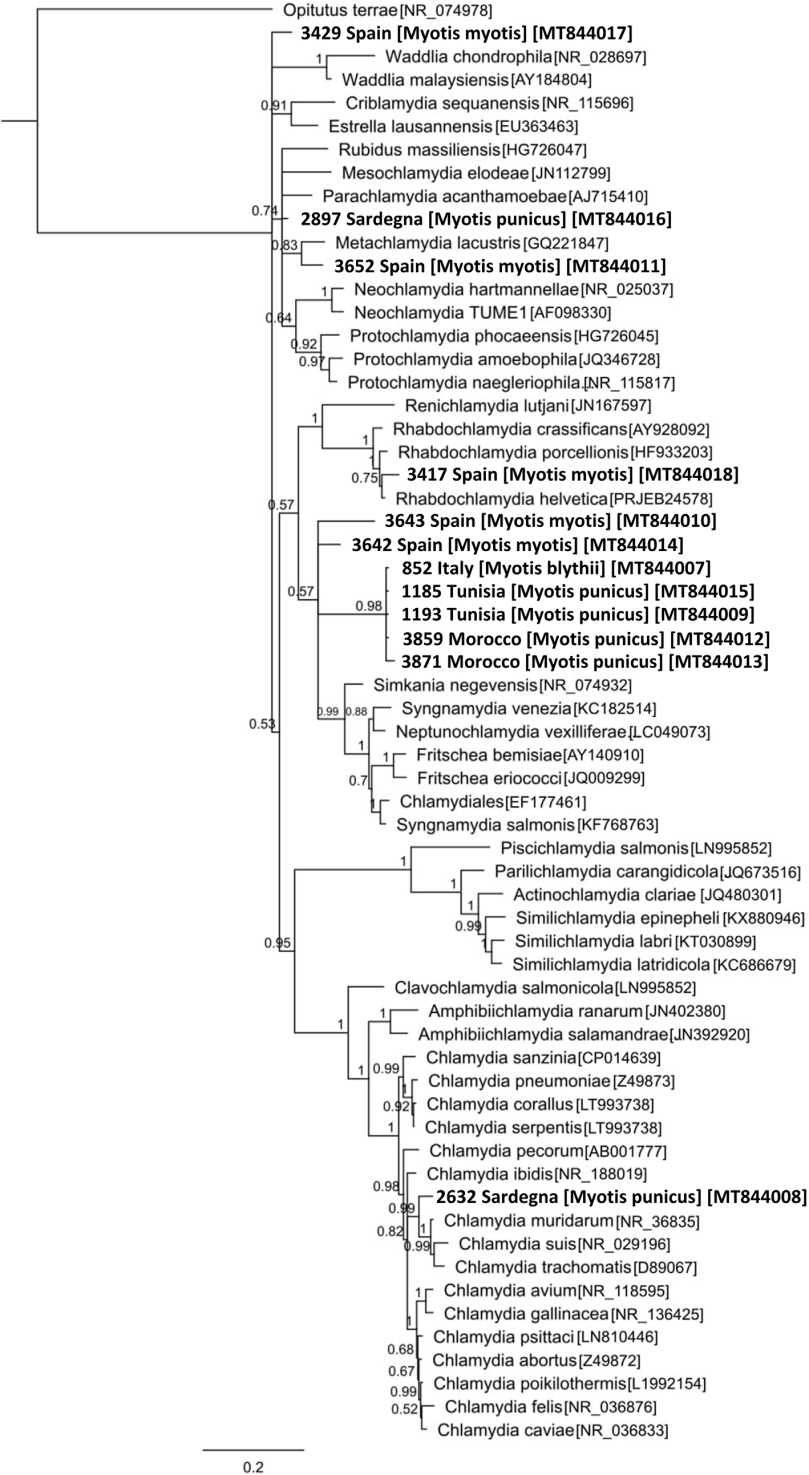
Phylogenetic Bayesian consensus tree of the *Spinturnix* sequences of this study (in bold) along with Chlamydiales-reference and outgroup sequences (all with their GenBank accession numbers) with the posterior probabilities of clades and the branch length for 16S rRNA. Only posterior probabilities of more than 50% are shown in the tree. In addition to their reference code, we also added the collection site and the bat host species of each *S. myoti* sequence.

In accordance with the BLAST analysis, the Bayesian phylogenetic tree revealed that most of the sequences that showed BLAST hit identities smaller than 92.5% were grouped together with a high posterior probability score and seem to form a previously unknown family of Chlamydiales (Fig. 2).

## Discussion

*Chlamydia* and *Chlamydia*-like organisms have recently been found in bat samples suggesting that bats may play a role as reservoirs for members of the Chlamydiae phylum [13, 29, 41]. However, the way bats become infected by these Chlamydiae is unclear. Here we found a high prevalence (57.95%) of Chlamydiae in *Spinturnix myoti*, an obligate, ectoparasitic mite species of mouse-eared bats (*Myotis* spp.), suggesting that mites may play a role as reservoirs or vectors. In addition, sequencing and phylogenetic analysis revealed that *S. myoti* mites harbor Chlamydiales from several families including a new family-level lineage, suggesting that the diversity of the Chlamydiales order is underestimated.

Although our knowledge of the epidemiology of the Chlamydiaceae family has increased rapidly due to their zoonotic potential, our understanding is still scarce concerning the ecology, diversity and epidemiology of *Chlamydia*-like organisms. CLOs are able to infect several organisms including humans [24, 25, 55], but transmission routes remain unknown. Previous studies have highlighted that amoebae may be reservoirs and dispersal vectors of different CLOs species, especially Parachlamydiaceae and Criblamydiaceae [30, 56]. Here, in addition to previous work done on ticks and fleas [9, 17, 28, 43], we confirmed that ectoparasites such as *Spinturnix* mites may play a role in the transmission of Chlamydiae. However, whether ectoparasites can effectively transmit Chlamydiae is not known and further studies are needed. A previous study showed that *Bartonella* infection found in humans was closely related to infections found in our target species *S. myoti*, suggesting that bat-associated bacterial pathogens can infect humans [53]. Additionally, other bacterial pathogens or possibly symbionts have been detected from mites, including *Spinturnix* spp., such as *Anaplasma* spp., *Bartonella* spp., *Rickettsia* spp. and *Spiroplasma* sp. [31, 45, 53].

Vector feeding preference is one of the most important components determining the distribution of diseases. Vectors that are specialized to take their blood meal from closely related hosts limit the distribution of the microbes they host to regions where they are present. On the other hand, vectors that are more generalists, i.e. feeding on distantly related host species, may favor the spread of pathogenic microbes they can transmit, possibly extending them to new host species. *Spinturnix* mites are known to display different levels of host specificity, ranging from one to several usually closely related bat species [1, 7, 8, 12]. Thus, they may play a major role in the distribution and/or maintenance of Chlamydiae bacteria within and between bat populations. In particular, *S. myoti* is rather specific to mouse-eared bats [57] but can disperse between closely-related species or by accidental transfer when these are in close contact. Importantly, while *M. punicus* are geographically localized in North Africa, Sardinia, Corsica and Malta [33], *M. blythii* and *M. myotis* are mainly found in continental Europe, sometimes forming mixed nursery colonies [12]. Despite the potential close contact between *M. blythii* and *M. myotis*, the Chlamydiae prevalence was significantly lower in the *Spinturnix* mites found on *M. blythii*, which might suggest that *M. blythii* is less vulnerable to Chlamydiae infection than *M. myotis*, and thus that *M. blythii* may have a lower reservoir potential for Chlamydiae. In addition, the prevalence in *Spinturnix* was significantly higher when *M. myotis* were living without any other bat species rather than when living in sympatry with *M. blythii*. This difference in prevalence between allopatric and sympatric *M. myotis* may reflect a dilution effect with *M. blythii* playing a role as an incompetent host. However, our results revealed that 20% of *S. myoti* collected on *M. blythii* were harboring Chlamydiae. A possibility may be that these infected *S. myoti* collected on *M. blythii* acquired their infection on *M. myotis* first before switching host. Spinturnicid mites are known to often switch between their hosts [23] and may thus carry their Chlamydiae from one host to another. However, since in this study *S. myoti* were collected on *M. blythii* only found in sympatry with *M. myotis*, this hypothesis cannot be confirmed. Further studies are thus needed to better understand the reservoir potential of bats for Chlamydiae, since it seems to be species-dependent, with *M. blythii* exhibiting at least a lower competence for Chlamydiae than *M. myotis*.

Altogether, these results indicate that *S. myoti* may contribute to the transmission and maintenance of Chlamydiae between bat species. The prevalence in *S. myoti* decreased when there is more than one host bat species, which indicates that the Chlamydiae may be distributed between the different bat species by these mites. In addition, our novel study showed that Chlamydiae infection in *Spinturnix* might be geographically variable depending of the bat species. While no difference between collection sites was found for *M. punicus* and *M. blythii*, there was a significant effect of the collection sites for *M. myotis*. However, these results may reflect the fact that the *Spinturnix* were collected on *M. myotis* living both in sympatry or allopatry and therefore deserve further investigations.

Although *Chlamydia* and *Chlamydia*-like bacteria have been detected in many different environmental samples and organisms, the diversity of the Chlamydiae phylum is highly underestimated. Most studies have shown that both environmental and organismal samples contain Chlamydiae that certainly represent new family-level lineages, thus new Chlamydiae species [28, 29, 42, 43, 55, 58], which our study also confirmed. While *S. myoti* harbored Chlamydiae from several families, the BLAST analysis revealed that more than half of the sequences were not attributable to a known family-level lineage, suggesting they belong to a new Chlamydiales family that may be specific to *S. myoti* or to bats. Of note, an *S. myoti* DNA sample was also documented positive for a Chlamydiaceae, which appeared to be closely related to *C. muridarum* found in small mammals, including rodents (Rodentia: Muridae). To our knowledge, this is only the second time that a member of the Chlamydiaceae family has been discovered in an arthropod. Additionally, *Chlamydia psittaci* has previously been isolated in *Dermanyssus gallinae*, a mite of canaries [14].

In conclusion, our results suggest that the ectoparasite *S. myoti* may play a role as a vector of Chlamydiae, since we found a high prevalence of these strict intracellular bacteria. Our findings highlight the limited knowledge about the ecology and epidemiology of Chlamydiae and the need for further investigations. Hence, as Chlamydiae may potentially impact human and animal health, future studies should focus on the understanding of the maintenance and transmission of this bacteria in bats. Furthermore, other obligate ectoparasites, such as bat fleas (Siphonaptera: Ischnopsyllidae), bat bugs (Hemiptera: Cimicidae and Polyctenidae) and bat flies (Diptera: Nycteribiidae and Streblidae) may also potentially act as reservoirs or vectors of a wide range of bacteria [6, 38, 51, 52], including Chlamydiae.
